# Supplementary material for: Aiolos restricts the generation of antigen-inexperienced, virtual memory CD8+ T cells
Source: bioRxiv. 2025 Jun 16:2025.06.11.659122. Preprint. [Version 1] doi: 10.1101/2025.06.11.659122 (PMC12262803; doi:10.1101/2025.06.11.659122)
Supplement: Supplement 1 [file media-1.pdf]

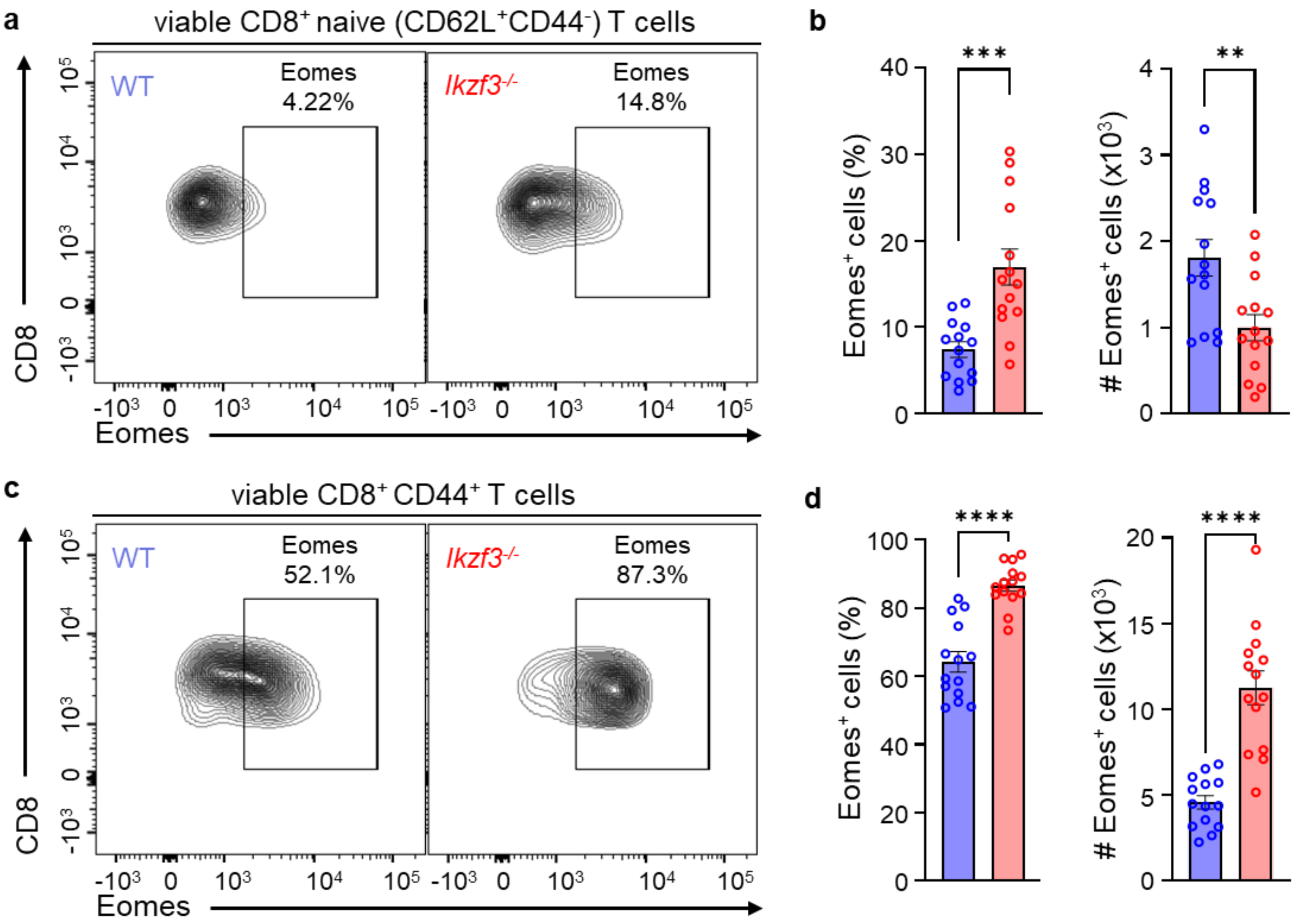

Supplementary figure 1: Eomes and Aiolos expression inversely correlate in CD8⁺ T cells. Flow cytometry analysis of Eomes expression in CD8⁺ naive (CD62L⁺CD44⁻) and activated (CD44⁺) T cells from the spleens of uninfected WT and *Ikzf3*⁻/⁻ mice. Representative flow cytometry contour plots and corresponding frequency and numbers (#, normalized to 6 x 10^5 events) of Eomes-expressing (**a-b**) naive and (**c-d**) effector CD8⁺ T cells. Data shown for 4 independent experiments, n=14, mean ± SEM, unpaired Student's t-test, **p≤0.01, ***p≤0.001, ****p≤0.0001.

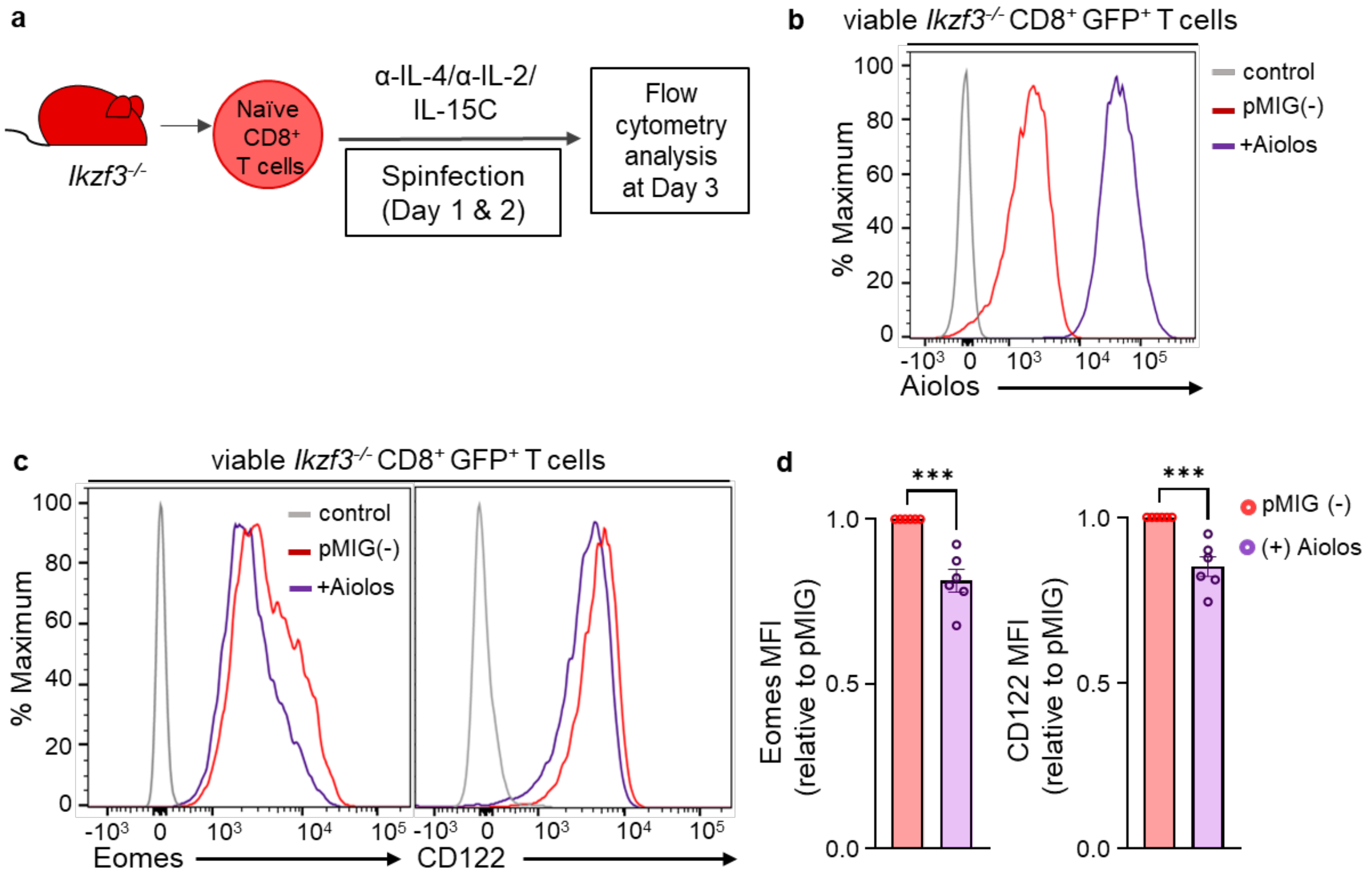

Supplementary figure 2: Aiolos overexpression results in decreased Eomes and CD122 expression in Aiolos-deficient CD8$^+$ T cells.

(**a**) Schematic of overexpression of Aiolos in Aiolos-deficient CD8$^+$ T cells. Naive CD8$^+$ T cells isolated from the spleens of *Ikzf3*$^{-/-}$ mice were spinfected with viral supernatants for Aiolos overexpression. (**b**) Representative histogram overlay confirming Aiolos overexpression in Aiolos-deficient CD8$^+$ T cells. (**c**) Histogram overlay for Eomes and CD122 and (**d**) associated MFI fold change data following the overexpression of Aiolos in Aiolos-deficient CD8$^+$ T cells *in vitro*. Data shown for 6 independent experiments, n=6, mean ± SEM, unpaired Student's t-test, ***p≤0.001.

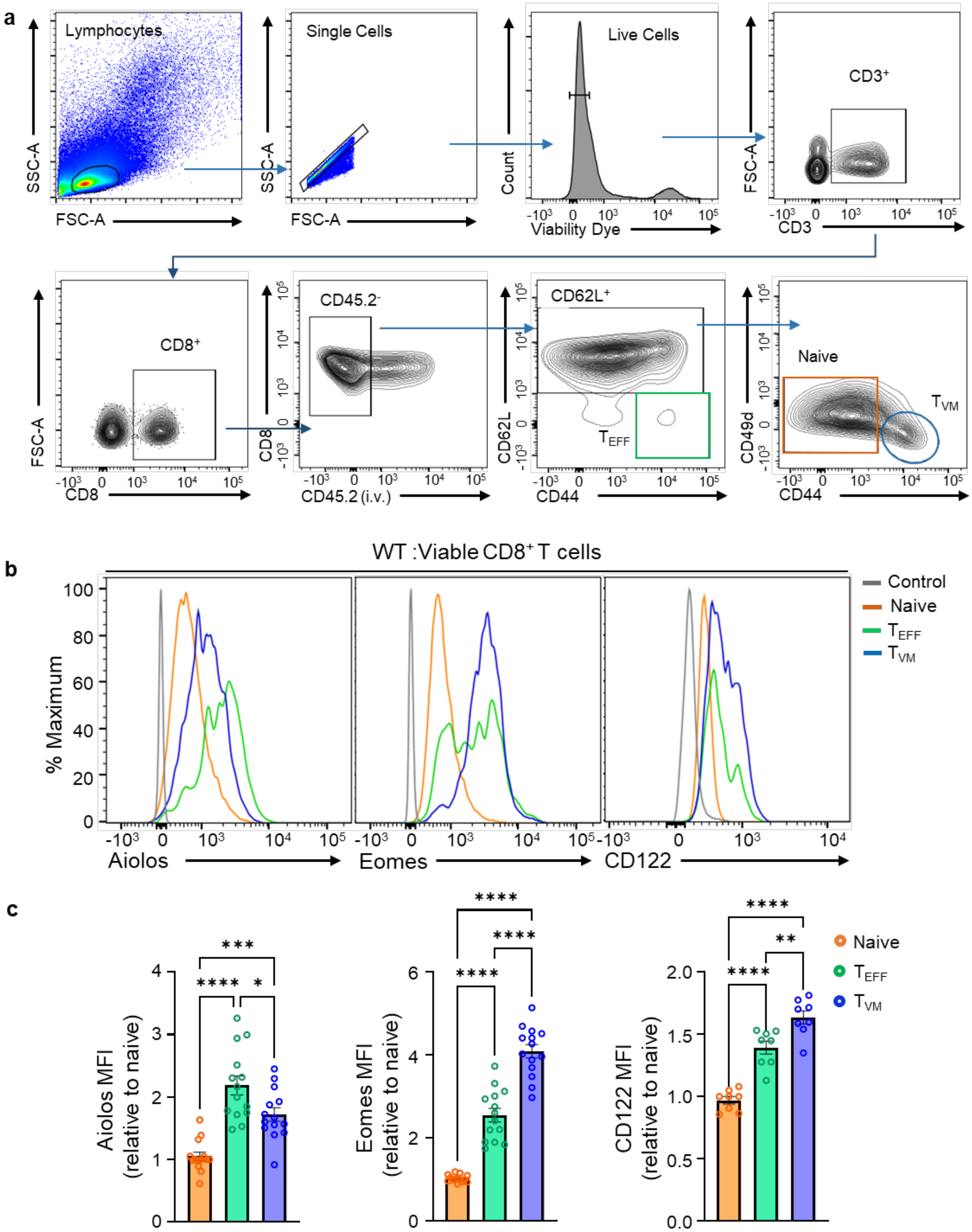

a
Lymphocytes
Single Cells
Live Cells
CD3+
SSC-A
FSC-A
Count
Viability Dye
CD3
CD8+
CD45.2-
CD62L+
Naive
TEFF
TVM
CD8
CD45.2 (i.v.)
CD62L
CD44
CD49d
b
WT :Viable CD8+ T cells
% Maximum
Aiolos
Eomes
CD122
Control
Naive
TEFF
TVM
c
Aiolos MFI (relative to naive)
Eomes MFI (relative to naive)
CD122 MFI (relative to naive)
Naive
TEFF
TVM

Supplementary Figure 3: Representative gating strategy and expression of Aiolos, Eomes, and CD122 in different $CD8^+$ T cell subsets.

(**a**) Gating strategy for $CD8^+$ T cells from uninfected or infected WT and *Ikzf3*$^{-/-}$ mice. Mice were retro-orbitally injected with PE-labeled anti-CD45.2 antibody prior to euthanasia to distinguish circulating (CD45.2$^{(i.v.\ Pos)}$) cells from resident (CD45.2$^{(i.v.\ Neg)}$) cells. $CD8^+$ naive, effector (T_{EFF}) and virtual memory (T_{VM}) cells were distinguished using CD62L, CD44, and CD49d markers as: Naive ($CD62L^+CD44^-CD49d^-$), effector ($CD62L^-CD44^+$), and T_{VM} ($CD62L^+CD44^+CD49d^{Lo}$). (**b-c**) Comparison of expression of Aiolos, Eomes, and CD122 across different $CD8^+$ T cell subsets in the spleens of uninfected WT mice. (**b**) Representative histogram overlays and (**c**) corresponding MFI fold changes for indicated proteins on naive, T_{EFF}, and T_{VM} cells. Data shown for 2-4 independent experiments, n=14 (for Aiolos and Eomes) and n=8 (for CD122), mean ± SEM; One-way ANOVA, Tukey's multiple comparisons test, *p≤0.05, **p≤0.01, ***p≤0.001 and ****p≤0.0001.

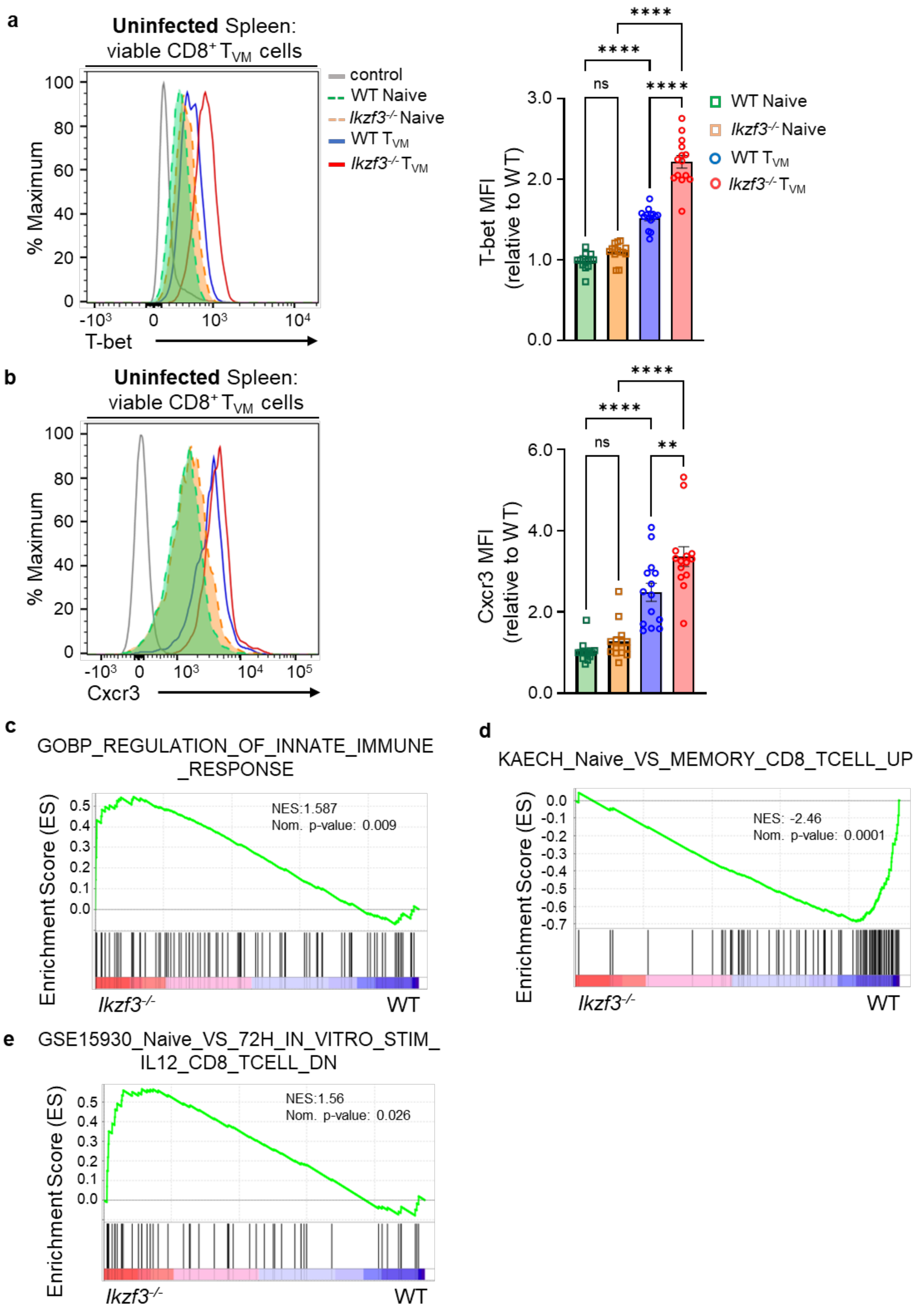
a
Uninfected Spleen: viable CD8+ TVM cells
% Maximum
T-bet
control
WT Naive
Ikzf3-/- Naive
WT TVM
Ikzf3-/- TVM
T-bet MFI (relative to WT)
ns
****
WT Naive
Ikzf3-/- Naive
WT TVM
Ikzf3-/- TVM
b
Uninfected Spleen: viable CD8+ TVM cells
% Maximum
Cxcr3
Cxcr3 MFI (relative to WT)
ns
**
****
c
GOBP_REGULATION_OF_INNATE_IMMUNE_RESPONSE
Enrichment Score (ES)
NES:1.587
Nom. p-value: 0.009
Ikzf3-/-
WT
d
KAECH_Naive_VS_MEMORY_CD8_TCELL_UP
Enrichment Score (ES)
NES: -2.46
Nom. p-value: 0.0001
Ikzf3-/-
WT
e
GSE15930_Naive_VS_72H_IN_VITRO_STIM_IL12_CD8_TCELL_DN
Enrichment Score (ES)
NES:1.56
Nom. p-value: 0.026
Ikzf3-/-
WT

Supplementary Figure 4: Aiolos suppresses T-bet and CXCR3 expression in T_{VM} cells and regulates various gene pathways.

(**a-b**) Representative histogram overlays and associated median fluorescence intensity (MFI) fold change for (**a**) T-bet and (**b**) CXCR3 expression in the indicated cell types, relative to WT naive $CD8^+$ T cells. Data shown for 4 independent experiments, n=14, mean ± SEM; two-way ANOVA with Tukey's multiple comparisons test, **p≤0.01, ****p≤0.0001. (**c-e**) Enrichment plots for indicated gene sets generated using the GSEA software for comparison against 'gene ontology', and 'immunological signature' gene sets of the pre-ranked genes.

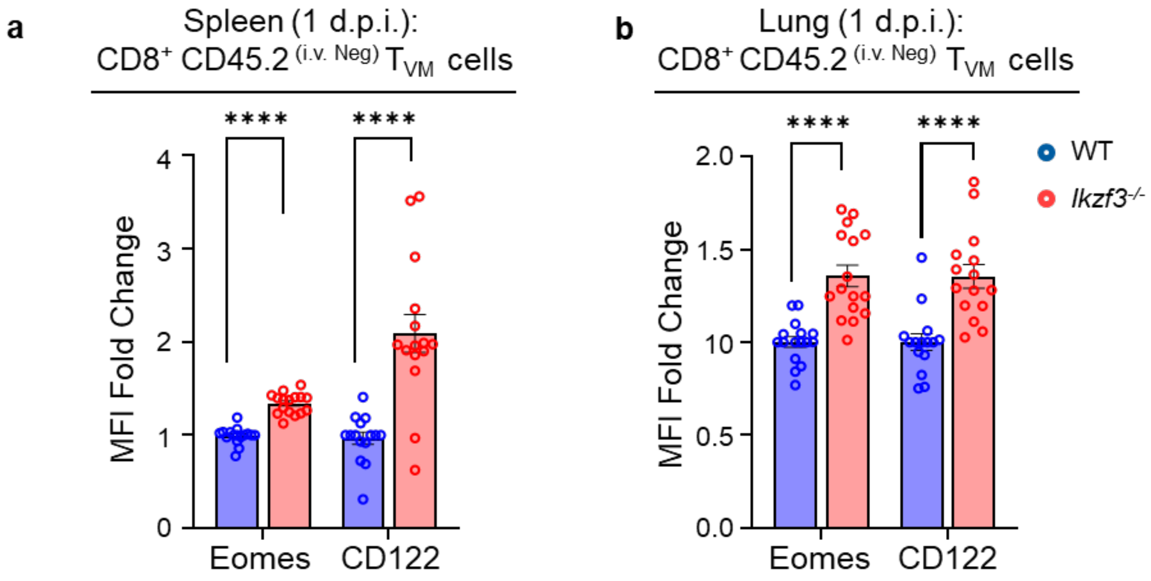

Supplementary Figure 5: Aiolos suppresses the expression of key T_{VM} regulatory factors.

Median fluorescence intensity (MFI) fold change for Eomes and CD122 expression relative to WT in $CD8^+$ T_{VM} cells from (**a**) the spleen and (**b**) lungs of WT vs. Aiolos-deficient mice 1 day following influenza virus challenge. Data representative of 5 independent experiments, n=14-16, mean ± SEM; unpaired Student's t-test, ****$p \leq 0.0001$.
